# Supplementary material for: The Influence of a Polyphenol-Rich Red Berry Fruit Juice on Recovery Process and Leg Strength Capacity after Six Days of Intensive Endurance Exercise in Recreational Endurance Athletes
Source: Nutrients. 2024 May 9;16(10):1428. doi: 10.3390/nu16101428 (PMC11124493; doi:10.3390/nu16101428)
Supplement: Supplementary file 1 [file nutrients-16-01428-s001.zip › nutrients-2944271-supplementary.pdf]

Supplementary Materials

Table S1: Measurement parameters and ingredients of the placebo and red fruit juice drink.

parameter	amount in Placebo	amount in juice (lot 1)	amount in juice (lot 2)
specific gravity [kg/L]	1,04867	1,0614	1,06129
Brix [°Bx]	12,0	15,22	15,45
conductance	2060	2380	2260
extract [g/L]	126,3	159,6	159
sugar-free extract [g/L]	18,2	40,7	41,9
sugar [g/L]	108,1	118,9	117,1
glucose [g/L]	52,4	58,4	56,3
fructose [g/L]	55,7	60,6	58,6
succrose [g/L]	< 0,5	< 0,5	2,3
glucose/fructose-ratio	0,94	0,96	0,96
pH-value	3,29	3,42	3,34
total acid pH 8,1 cal. Cs [g/L]	6,47	7,24	7,03
ascorbic acid [mg/L]	n.n.	5	7
volatile acid [g/L]	0,02	0,04	0,04
L-malic acid [g/L]	< 0,05	2,91	2,86
citric acid [g/L]	6,76	4,45	4,85
**total phenols [mg/L]**	**91**	**4052**	**4159**
zinc [mg/L]	< 0,1	0,6	0,6
ferrum [mg/L]	< 0,1	1,5	1,4
potassium [mg/L]	1498	1429	1467
calcium	< 1	158	164
magnesium [mg/L]	< 1	97	94
copper [mg/L]	< 0,1	0,1	0,1

Table S2: Overview of the six-day endurance training protocol documentation.

ID	Training time juice [min]	Training km juice [km]	ER km juice [km]	Interval km juice [km]	Pace/ overall juice [min/km]	Training time placebo [min]	Training km placebo [km]	ER km placebo [km]	Interval km placebo [km]	Pace / overall placebo [min/km]
**P01**	210.00	38.02	17.98	20.04	5.52	210.00	36.32	17.34	18.98	5.78
**P02**	210.00	42.42	18.87	23.55	4.95	210.00	39.70	17.78	21.92	5.29
**P03**	210.00	56.65	21.41	35.24	3.71	210.00	51.66	21.33	30.33	4.07
**P04**	210.00	51.75	23.92	27.83	4.06	210.00	52.55	24.33	28.22	4.00
**P05**	210.00	61.88	22.51	39.37	3.39	210.00	59.38	22.64	36.74	3.54
**P06**	210.00	52.70	20.28	32.42	3.98	210.00	46.44	20.03	26.41	4.52
**P07**	210.00	41.15	17.55	23.60	5.10	210.00	40.13	17.57	22.56	5.23
**P08**	210.00	42.75	17.77	24.98	4.91	210.00	44.87	18.98	25.89	4.68
**P09**	210.00	39.86	18.90	20.96	5.91	210.00	38.10	18.98	19.12	5.51
**P10**	210.00	64.02	22.05	41.97	3.28	210.00	67.81	23.53	44.28	3.10
**P11**	210.00	48.72	21.21	27.51	4.31	210.00	50.37	23.25	27.12	4.17
**P12**	210.00	44.24	18.99	25.25	4.75	210.00	49.04	23.10	25.94	4.28
**P13**	210.00	41.65	19.31	22.34	5.04	210.00	42.28	20.74	21.54	4.97
**P14**	210.00	40.23	18.25	21.98	5.22	210.00	44.11	21.13	22.98	4.76
**P15**	210.00	39.46	18.12	21.34	5.32	210.00	40.30	19.32	20.98	5.21
**P16**	210.00	65.90	26.38	39.52	3.19	210.00	64.22	24.12	40.10	3.27
**P17**	210.00	40.56	18.10	22.46	5.18	210.00	38.58	18.49	20.09	5.44
**P18**	210.00	42.31	18.03	24.28	4.96	210.00	42.63	18.04	24.59	4.93
**mean**	210.00	47.76	20.09	27.67	4.58	210.00	47.40	20.74	26.66	4.58
**SD**	0.00	9.34	2.49	7.24	0.84	0.00	9.33	2.40	7.42	0.81

Table S3: Strength test and blood parameters before and after a 6-day intensive endurance training time interval for the placebo and juice interventions.

			**JUICE**			**PLACEBO**			***p*-value**			
**Parameter**	Unit	N	**T0**	**T1**	**Δ**	**T0**	**T1**	**Δ**	**time**	**group**	**time x group**	**time**
**CK**	[U/L]<br>Log10	17	199.24 ± 149.60<br>2.20 ± 0.07	316.35 ± 159.29*<br>2.44 ± 0.06	**117.12 ± 191.75**<br>0,24 ± 0,07	215.18 ± 118.25<br>2.26 ± 0.07	379.53 ± 221.60*<br>2.51 ± 0.07	**164.35 ± 267.00**<br>0.25 ± 0.07	**0.001**	0.371	0.976	**0.170**
**oxLDL**	[U/L]<br>Log10	18	64 ± 51.40<br>1.66 ± 0.09	68.78 ± 58.70<br>1.67 ± 0.10	4.78 ± 37.52<br>0,01 ± 0,07	60.39 ± 61.37<br>1.59 ± 0.11	83.22 ± 92.08<br>1.71 ± 0.11	22.83 ± 71.56<br>0.12 ± 0.07	0.120	0.632	0.354	0.040
**est. 1 RM**	[kg]	18	104.22 ± 24.21	105.56 ± 24.54	**1.34 ± 9.26**	104.28 ± 22.00	100.95 ± 17.74	**-3.33 ± 11.49**	0.168	0.988	0.172	0.030
**rel. est. 1 RM**	[kg/kg KG]	18	1.40 ± 0.26	1,44 ± 0.26	0.04 ± 0.14	1.41 ± 0.20	1.38 ± 0.20	-0.03 ± 0.17	0.438	0.941	0.269	0.010
**IL-6**	[pg/ml]<br>Log10	18[a]	0.77 ± 0.58<br>-0.26 ± 0.12	0.74 ± 0.64<br>-0.30 ± 0.14	-0.1 ± 0.55<br>-0.01 ± 0.12	1.20 ± 1.27<br>-0.50 ± 0.35	1.09 ± 1.54<br>-0.25 ± 0.14	0.17 ± 1.24<br>0.14 ± 0.19	0.256	0.383	0.278	0.040
**IL-10**	[pg/ml]<br>Log10	18[a]	10.18 ± 12.26<br>0.81 ± 0.11	6.65 ± 5.06<br>0.62 ± 0.13	-2.80 ± 9.67<br>-0.13 ± 0.11	7.35 ± 6.07<br>0.67 ± 0.13	6.86 ± 5.31<br>0.61 ± 0.14	-0.004 ± 3.76<br>-0.01 ± 0.09	0.737	0.434	0.189	0.000

[a]In the evaluation of cytokines, there are 30 missing values for IL-6 and 17 missing values for IL-10. CK = Creatin kinase, oxLDL = oxidized low-density lipoprotein, est. 1 RM = estimated 1 repetition maximum, rel. est. 1 RM = relative estimated 1 repetition maximum, IL-6 = Interleukin 6, IL-10 = Interleukin.
